# Elevation Filters Lizard Species Occurrences and Assemblages in Chitwan Annapurna Landscape, Nepal

**DOI:** 10.1002/ece3.73732

**Published:** 2026-06-01

**Authors:** Bishnu Prasad Bhattarai, Sandeep Regmi, Bishnu Aryal, Pradip Kandel, Jagan Nath Adhikari

**Affiliations:** ^1^ Central Department of Zoology, Institute of Science and Technology Tribhuvan University Kirtipur, Kathmandu Nepal; ^2^ Southeast Asia Biodiversity Research Institute, Chinese Academy of Sciences and Center for Integrative Conservation, Xishuangbanna Tropical Botanical Garden Chinese Academy of Sciences Mengla Yunnan China; ^3^ The Himalayan Conservancy Kirtipur, Kathmandu Nepal; ^4^ University of Chinese Academy of Sciences Beijing China; ^5^ Department of Zoology Birendra Multiple Campus Bharatpur, Chitwan Nepal

**Keywords:** Central Himalaya, CHAL, elevational gradient, herpetofauna, lizards, species turnover

## Abstract

Understanding elevational patterns of biodiversity is essential for conservation planning. However, the spatial distribution pattern of lizards in the central Himalayan elevation gradient has rarely been investigated or is mostly from outdated literature. We carried out a study on the occurrence and associated factors of lizard species across the elevation gradients of Chitwan Annapurna Landscape (CHAL) during 2018–2019. This study was carried out by visual encounter survey along the transects (100 × 5 m) and examined the patterns of lizard species richness. We reported 23 species of lizards across our study where most observed species were *Calotes versicolor* (*n* = 405), followed by *Hemidactylus flaviviridis* (*n* = 250), and *Laudakia tuberculata* (*n* = 128), whereas the least observed species were *Phrynocephalus theobaldi, Asymblepharus nepalensis, and Lygosoma albopunctata* which were observed only twice. We observed that species richness varied significantly with elevation (Kruskal–Wallis *χ* ^2^ = 41.29, *p* = 0.0076), although the monotonic relationship was weak (Spearman's *ρ* = 0.071, *p* = 0.031). Beta diversity was mostly driven by species turnover rather than nestedness. We observed significant non‐linear impact of elevation on gamma diversity (edf = 2.92, *p* < 0.001), where terrain ruggedness positively influenced the lizard species richness (*β* = 0.383, *p* < 0.001) and canopy cover had a negative effect (*β* = −0.154, *p* = 0.033). Our study demonstrates that the lizard diversity in CHAL is structured by non‐linear elevational dynamics and microhabitat heterogeneity. Also, we found a strong turnover of species across elevation bands. These results emphasize the ecological need to consider ecotonal zones and topographic complexity to conserve and maintain reptilian diversity in the Nepal Himalayas. Further, detailed and long‐term studies are needed to develop conservation initiatives and fill gaps in knowledge of lizards in the human‐dominated fragmented landscape of the Central Himalayas.

## Graphical abstract

We investigated lizard species richness,assemblage patterns, and environmental drivers along the elevational gradient in the Chitwan Annapurna Landscape during 2018–2019. We recorded 23 lizard species and found that species richness varied significantly and non‐linearly with elevation, peaking at mid elevation. Species turnover dominated beta diversity, and elevation, terrain ruggedness, and canopy cover were the key predictors of diversity patterns.

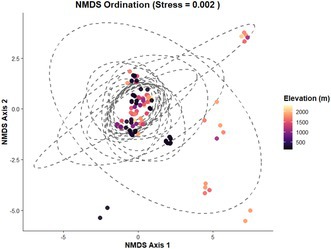

## 1 Introduction

Biodiversity in a landscape is not only a high‐priority scientific theme, but also a key indicator of ecosystem health and resilience (Katayama et al. 2014; Körner 2024). Landscapes with varied terrain and elevation typically exhibit greater environmental heterogeneity and host higher biodiversity, primarily due to the presence of multiple climatic zones and habitat types that support diverse ecological niches (Stein et al. 2014; Wan et al. 2023). However, global biodiversity is currently undergoing rapid decline partly due to a range of anthropogenic drivers such as habitat loss, climate change, and overexploitation (Mazor et al. 2018; Prakash and Verma 2022). This trend underscores the urgent need to identify the environmental and anthropogenic factors that shape species occurrence and richness patterns to inform targeted conservation and management strategies—both globally and in Nepal (Willis and Whittaker 2002; Sanders and Rahbek 2012).

The Nepal Himalaya, one of the most prominent global biodiversity hotspots, exhibits one of the extreme elevational gradients encompassing a broad range of climatic zones—from tropical lowlands to alpine and nival regions—within a relatively short vertical distance (Zhao et al. 2022, 2023). Elevation is a well‐established determinant of species richness and composition, and a mid‐elevation peak in species diversity is commonly observed pattern across various taxa and geographic regions (Stevens 1992; Colwell and Lees 2000; McCain 2004). However, research conducted explicitly on herpetofauna highlight monotonic pattern in richness in relation to the elevation gradient (Fu et al. 2007; Chettri et al. 2010; Shrestha and Shah 2017; Bhattarai 2020; Jins et al. 2021). Nevertheless, such elevational patterns can vary among vertebrate groups and regions (Chettri et al. 2010; McCain 2010) and remain poorly explored for many vertebrate groups in Nepal, particularly reptiles (Rai et al. 2022). For lizards specifically, the information on how species distribution varies across elevational gradients is still scarce.

Nepal harbors a diverse assemblage of herpetofauna (Schleich and Kästle 2002; Shah and Tiwari 2004) consisting of 57 species of amphibians and 143 species of reptiles (Rai et al. 2022). Existing research on reptiles in Nepal has largely focused on species checklists (Schleich and Kästle 2002; Shah and Tiwari 2004; Rai et al. 2022) or concentrated in small area and single species (Bhattarai 2020; Rawat et al. 2020). More broadly, studies on herpetofauna along elevational gradients have reported variable richness patterns with several documenting monotonic declines in species richness with increasing elevation (Fu et al. 2007; Chettri et al. 2010), indicating that elevational patterns remain context dependent rather than being universally consistent. However, such relationships remain poorly documented for reptiles in Nepal, particularly lizards (Gautam 2018; Rawat et al. 2020). This reflects a broader global pattern where reptiles have historically received comparatively less research attention and conservation investment than other vertebrate groups, resulting in major ecological, distributional, and conservation data gaps (Caetano et al. 2022; Speight et al. 2025). As a consequence, most of the lizard species are categorized as data deficient by IUCN Red list of threatened species (Caetano et al. 2022).

The Chitwan Annapurna Landscape (CHAL) in the Central Himalayas of Nepal represents a biodiversity‐rich region with high topographic and climatic variation (Schleich and Kästle 2002; Shah and Tiwari 2004). Despite its ecological significance, CHAL is human‐dominated and highly fragmented (Adhikari et al. 2022, 2024). To the best of our knowledge, limited research has been conducted assessing the factors shaping lizard species richness and distribution in CHAL or more broadly in Nepal (Gautam 2018; Bhattarai 2020). As such, understanding the distribution patterns of these species and their underlying mechanisms along elevational gradient is necessary for conservation of lizards as well as overall biodiversity (Grytnes and Vetaas 2002; Bhattarai et al. 2004). Therefore, this study provides a valuable addition to literature and an important baseline in prioritizing areas for lizard species conservation planning (Hunter and Yonzon 1993). This study is the first systematic study on lizard species assemblage and compositional change along elevational gradient in the Central Himalayas of Nepal.

## 2 Materials and Methods

### 2.1 Study Area

**FIGURE 1:**
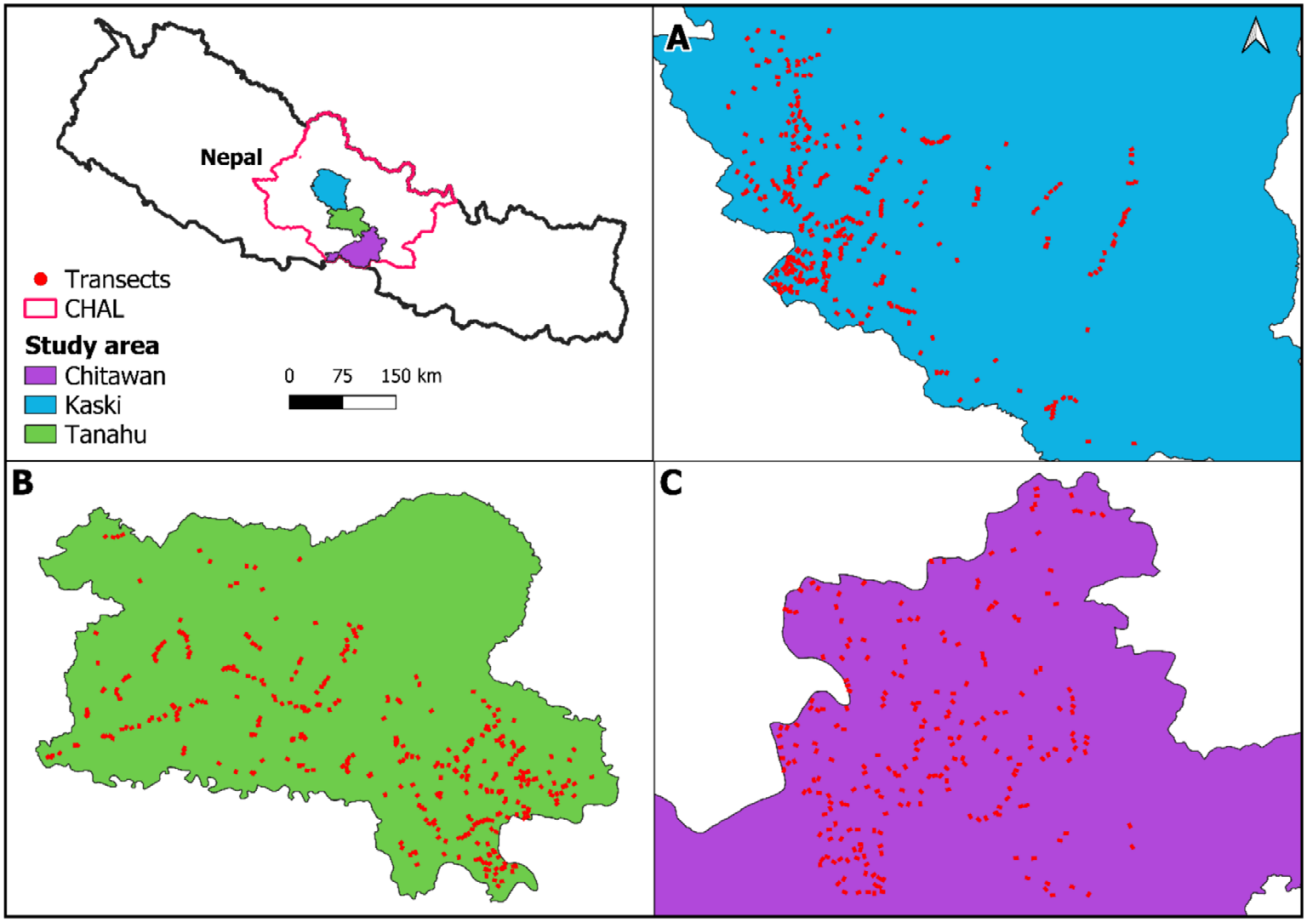
Map of study area showing study blocks (Block A: Chitwan, Block B: Tanahun, and Block C: Kaski) and study transects.

This study was conducted in the Chitwan‐Annapurna landscape (CHAL), located on the southern slope of the Central Himalayas in Nepal (Figure 1). The study covers two protected areas: Chitwan National Park and Annapurna Conservation Area along the elevation gradient. CHAL is characterized by rugged terrain with high seasonal climatic variability. The spatial change in climatic variation is strongly influenced by elevation gradient. Summer monsoonal rainfall from Bay of Bengal strongly affects the seasonal temperature and precipitation in this region (Bookhagen and Burbank 2010). The mean annual temperature varies between 7°C and 26°C, and precipitation mostly occurs (about 80%) during May to October (Vetaas 2000; DHM 2019). Climate factors also vary along the elevation gradient, with mean annual temperature declining linearly from 23.4°C to 7°C with elevation. In contrast, mean annual precipitation increases from 1000 to 2600 mm between the lowlands and the summit areas.

The elevation of CHAL ranges from around 164 m to above 8000 m. The lower regions experience tropical and subtropical climates, while the middle hills have temperate climates, and the higher mountain areas exhibit alpine and subalpine climate (Adhikari et al. 2022). This landscape harbors abundant biodiversity, encompassing three of the world's globally significant ecoregions: the Terai–duar Savanna and Grasslands, Himalayan Subtropical Broadleaf Forests, and Himalayan Sub‐tropical Pine Forest (Dinerstein et al. 2017) and two Ramsar sites (Beeshazari and associated lakes, Chitwan and Lake Clusters of Pokhara valley, Kaski) (NLCDC. 2020). This region serves as a crucial habitat for a wide variety of significant mammal species, avian species, reptiles and amphibians (herpetofauna), fish, as well as many small and large invertebrates (Bhuju et al. 2007; WWF 2013).

### 2.2 Data Collection

Lizard surveys were carried out by time‐constant visual encounter survey during the months of March to September (single season, single visit per transect). This period coincides with the monsoonal rainfall and breeding season of lizards (Schleich and Kästle 2002). Surveys were carried out during daytime along transects (100 × 10 m) between 7.00 and 19.00 h. For highly cryptic lizards such as skinks, we actively searched under logs, leaf litter, and rocks and scanned the sites. Loose barks of the trees were carefully removed to scan if there is a possible occurrence of any lizard species. Although the diurnal survey may under‐detect the nocturnal species, we believe that the combination of systematic scanning (e.g., search along line transects) and active microhabitat searches may potentially reduce the probability of missing such species. Systematic scanning involved slow, continuous walking along the transects for visual searches within the predefined transect belt on either side. We divided the study area into three sampling blocks based on administrative boundaries: Block A (Chitwan District), Block B (Tanahaun District), and Block C (Kaski District). We established and surveyed a total of 293 transects in Block A, 390 in Block B, and 302 in Block C covering an overall survey length of 98.5 km. The transects were established along elevation gradients, with each 100 m change treated as a sampling interval. A total of 23 elevation bands were sampled across the study area. Within these bands, transects were created with 65 transects per band on average, except for the 100–200 m elevation band, which contained 75 transects due to higher accessibility and sampling intensity in low‐elevation areas.

Each transect was surveyed by four observers for 30 min to maximize the detection probability and minimize the likelihood of missing individuals of lizards. Transects were placed at least 250 m apart to ensure spatial independence and were primarily located in proximity to human‐modified landscapes and settlements. For each transect, all the encountered lizard species were identified and recorded. The identification was done in the field with the help of field guide books (Schleich and Kästle 2002; Shah and Tiwari 2004). The individuals difficult to identify in the field were photographed and later compared with the field guide.

Environmental variables were compiled from multiple sources. Canopy cover and shrub cover percentage were extracted from global 10 m land cover data of sentinel‐2 satellite imagery (Karra et al. 2021). Similarly, invasive alien plant species cover was measured from the field by creating 10 m × 10 m plot at each species occurrence point. Mean annual temperature, mean annual precipitation, and minimum temperature of coldest month were obtained from bioclimatic variables available from Worldclim database (https://worldclim.org/). We used minimum temperature of coldest month as it captures extreme cold constraints that are ecologically important for ectotherms like lizards, particularly in Himalayan environments where cold tolerance can strongly influence distribution limits. Soil moisture was obtained from Copernicus land monitoring system, whereas terrain ruggedness index was calculated using a 90 m void filled digital elevation model from Shuttle Radar Topography Mission (SRTM).

### 2.3 Data Analysis

All statistical analyses were conducted in R software (R Core Team 2024). At first, species occurrence data were extracted from the full dataset and converted into transect × species presence by selecting columns corresponding to lizard taxa. Afterwards, the data were converted to presence‐absence format using the “decostand” function in the “vegan” package (Oksanen et al. 2013), and transects with zero species records were removed to avoid empty rows in multivariate analyses. Species richness at the transect level was calculated as the row‐wise sum of presences and used as a measure of alpha diversity. To examine broader elevational patterns while reducing fine‐scale stochastic variation, transects were stratified into fixed 100 m elevation intervals. For each band, gamma diversity was calculated as the total number of unique lizard species recorded across all transects within that interval. Midpoint elevation of each band was used as a continuous predictor in band‐level models. We measured the relationships between transect‐level alpha diversity and elevation using Spearman's rank correlation, while differences in richness among elevational bands were tested using the Kruskal–Wallis test. The identification of elevation bands that differ significantly from other bands was done using Dunn's test with Benjamini‐Hochberg correction.

We quantified compositional variation among transects through pairwise beta diversity using pairwise Sørensen dissimilarity. The total beta diversity was partitioned into turnover and nestedness components using the “betapart” package (Baselga et al. 2023) to distinguish species replacement from richness‐driven nestedness. For visualization, transect‐level dissimilarity values were summarized across 100 m elevational bands. Differences in lizard community composition along elevation were tested using permutational multivariate analysis of variance (PERMANOVA) based on Jaccard dissimilarity and 999 permutations, using transect elevation as the explanatory variable. To visualize the compositional structure in reduced ordination space, we used non‐metric multidimensional scaling (NMDS), and ordination fit was assessed using stress value.

To assess large‐scale richness patterns, we modeled gamma diversity across 100 m elevational bands using generalized additive models (GAMs) in the mgcv package. Since the species richness represented count data, models were fitted with a Poisson error distribution and log link. Elevation midpoint was included as a penalized smooth term with smoothing parameters estimated using restricted maximum likelihood (REML) to balance model fit and complexity. To account for unequal sampling effort among elevation bands, we used the natural logarithm of the number of transects per band as an offset term.

Environmental predictors were averaged across transects within each elevational band. Prior to modeling, correlation and collinearity among environmental variables was assessed using “vifcor” function in “usdm” package (Naimi 2015), applying thresholds of *r* > |0.70| and variance inflation factor (VIF) > 5. Seven variables were retained for the final analyses which included canopy cover, shrub cover, invasive alien plant species (IAPs) cover, terrain ruggedness, precipitation of driest month, minimum temperature of the coldest month, and soil moisture (Table 1). Model adequacy was evaluated using residual diagnostics and inspection of effective degrees of freedom to ensure appropriate smoothing and variance assumptions.

**TABLE 1:** Suitable variables for analysis of lizard species richness in CHAL after collinearity test using variance inflation factor (VIF).

Predictors	VIF
Tree canopy	1.96
Shrub cover	1.71
IAPs cover	1.86
Terrain ruggedness	1.59
Precipitation of driest month	1.65
Minimum temperature of the coldest month	3.34
Soil moisture	1.54

## 3 Results

We recorded a total of 938 occurrences belonging to 23 species of lizards in CHAL (Table 2). The most observed species were *Calotes versicolor* (*n* = 405) followed by *Hemidactylus flaviviridis* (*n* = 250) and *Laudakia tuberculata* (*n* = 128), whereas the least observed species were *Phrynocephalus theobaldi, Asymblepharus nepalensis*, *and Lygosoma albopunctata*, which were observed only two times.

**TABLE 2:** List of species of lizards reported in the study area.

SN	Family/common names	Zoological names	CHAL status	CHAL location	NRDB	CITES	IUCN	Elevation range
Family—Agamidae								
1	Changeable Lizard	*Calotes versicolor* (Daudin, 1802)	VC	CHAL	—	—	LC	157–2909
2	Large Mountain Lizard	*Japalura major* (Jerdon, 1870)	UC	Panchase	—	—	LC	1338–1724
3	Three‐keeled Forest Agama	*Japalura tricarinata* (Blyth, 1853)	VR	CHAL hills	—	—	LC	1457–1839
4	Kashmir Rock Agama	*Laudakia tuberculata* (Gray, 1827)	CO	CHAL hills	—	—	LC	151–2248
5	Theobald's Toad‐headed Agama	*Phrynocephalus theobaldi* Blyth, 1863	VR	Ghandruk	—	—	LC	1847–1900
Family—Gekkonidae								
6	Flat‐tailed House Gecko	*Hemidactylus platyurus* (Schneider, 1792)	Ra	CHAL hills	—	—	LC	1317–2328
7	Nepalese Rock Gecko	*Cyrtodactylus* sp.	VR	Panchase	—	—	DD	1075–1860
8	Spotted House Gecko	*Hemidactylus brookii* Gray, 1845	Ra	CHAL	—	—	LC	1101–2057
9	Yellow‐bellied House Gecko	*Hemidactylus flaviviridis* Rüppell, 1835	VC	CHAL	—	—	LC	148–2314
10	Common House Gecko	*Hemidactylus frenatus* Schlegel in Duméril & Bibron, 1836	UC	BCF	—	—	LC	162–634
11	Fox Gecko	*Hemidactylus garnotii* Duméril & Bibron, 1836	VR	CHAL hills	—	—	LC	1075–1860
Family—Scincidae								
12	Himalayan Ground Skink	*Asymblepharus himalayanus* (Günther, 1864)	VR	Ghandruk	—	—	LC	1549–1833
13	Nepal Ground Skink	Eremchenko & Helfenberger, 1998	VR	Landruk	—	—	DD	1672–1875
14	Bronze Mabuya	*Eutropis macularia* (Blyth, 1853)	UC	CHAL	—	—	LC	650–1408
15	Keeled Indian Mabuya	*Eutropis carinata* (Schneider, 1801)	UC	CHAL	—	—	LC	1258–2136
16	Striped Grass Mabuya	*Eutropis dissimilis* (Hallowell, 1857)	Ra	BCF	—	—	LC	176–1238
17	Spotted Forest Skink	*Sphenomorphus maculatus* (Blyth, 1853)	UC	CHAL	—	—	LC	257–2168
18	Common Dotted Garden Skink	*Lygosoma punctata* (Gmelin, 1799)	VR	BCF	—	—	LC	143–229
19	White‐spotted Supple Skink	*Lygosoma albopunctata* (Gray, 1846)	VR	BCF	—	—	LC	217–247
20	Large Ground Skink	*Scincella capitanea* Ouboter, 1986	UC	Panchase	—	—	LC	193–310
21	Sikkim Ground Skink	*Asymblepharus sikimmensis* (Blyth, 1854)	UC	CHAL	—	—	LC	1033–2155
Family—Varanidae								
22	Bengal Monitor Lizard	*Varanus bengalensis* (Daudin, 1802)	UC	CHAL	S	I	NT	179–1238
23	Yellow Monitor	*Varanus flavescens* (Hardwicke & Gray, 1827)	UC	CHAL	S	I	EN	176–383

*Note:* CHAL status of lizards was determined on the basis of their abundance (number of individuals). VC = very common (> 200 individuals), Co = common (50–200 individuals), UC = uncommon (10–49 individuals).

**FIGURE 2:**
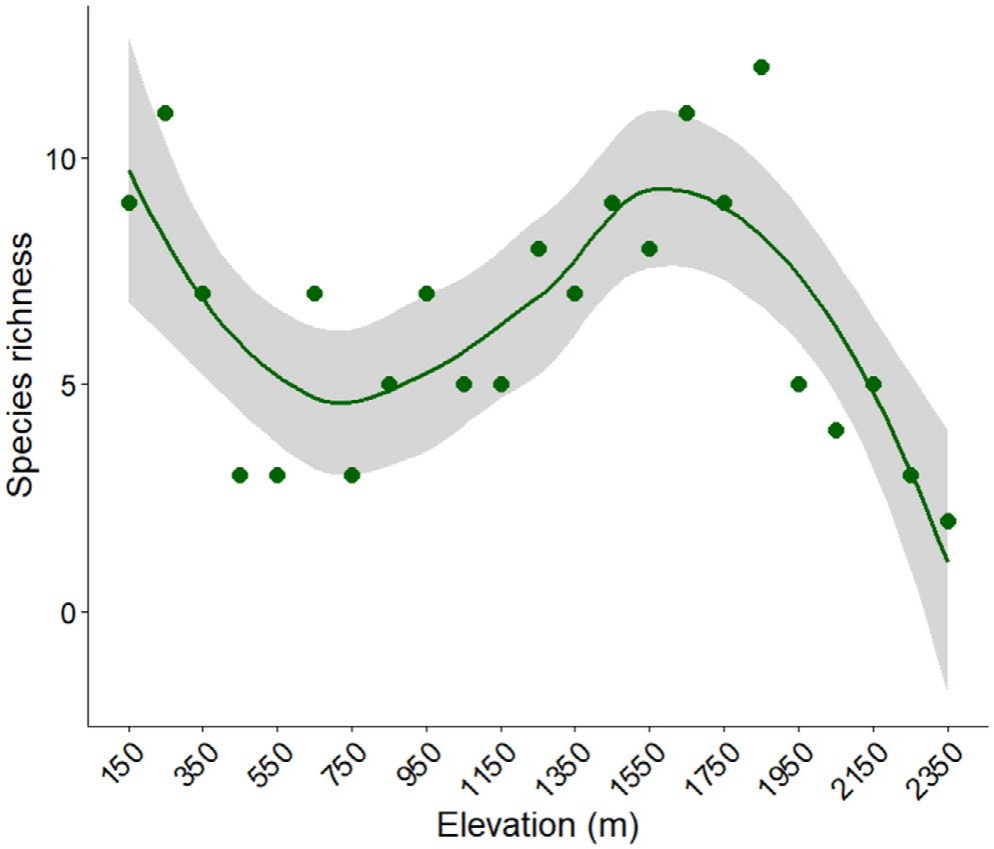
Effect of elevation on lizard species richness in CHAL.

Species richness (alpha diversity) varied significantly across the transects, with significant difference in mean site‐level richness (Kruskal–Wallis *χ* ^2^ = 41.29, df = 22, *p* = 0.0076; Figure 2). However, the strength of the monotonic relationship between elevation and species richness was comparatively weak. Spearman's rank correlation indicated a small but significant positive association between species richness and elevation (*ρ* = 0.071, *p* = 0.031) suggesting non‐linear variation in richness along elevation rather than a simple linear increase or decline. The post hoc examination showed that the elevation band 1500–1600 m differed significantly from other bands (*p* < 0.05), followed by 1600–1700 m band (*p* < 0.05).

**FIGURE 3:**
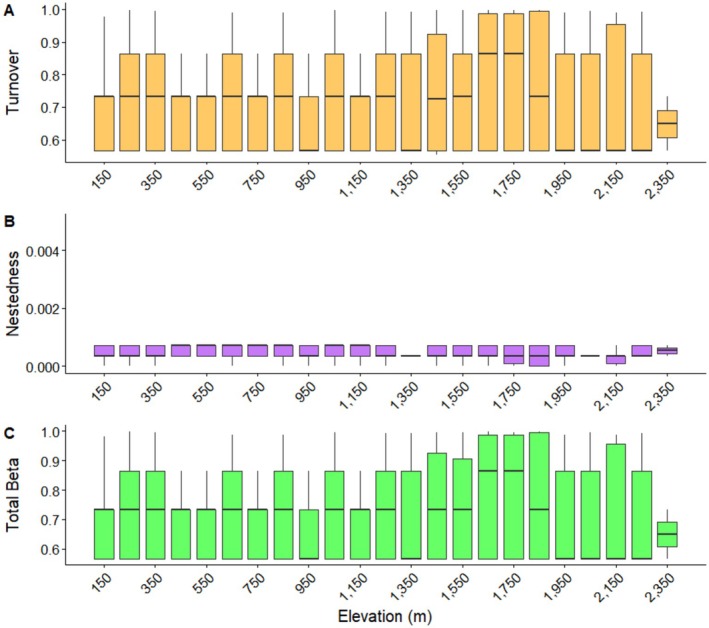
Beta partitioning of lizard species assemblage in CHAL. (A) Turnover, (B) Nestedness, (C) Total beta diversity.

Beta diversity partitioning using Sørensen dissimilarity showed that mean total beta diversity was high (*β*_sor = 0.721), and was almost entirely driven by species turnover (*β*_sim = 0.720), whereas the nestedness component was negligible (*β*_sne = 0.001; Figure 3). This indicates that compositional differences among sites were primarily due to species replacement rather than ordered species loss or gain along elevation. Boxplot patterns across elevation bands further supported strong turnover dynamics with minimal nested structuring.

**FIGURE 4:**
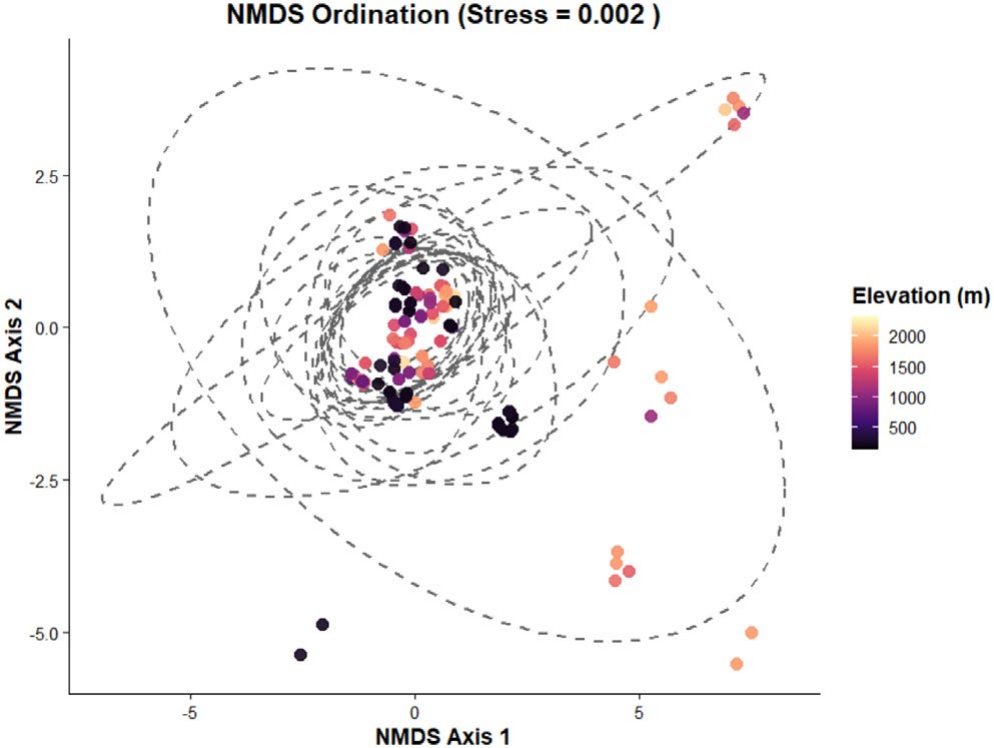
Ordination space showing gradual compositional shifts in lizard species richness along the elevational gradient.

PERMANOVA based on Jaccard distances detected a statistically significant effect of altitude on community composition (*F* = 9.22, *R* ^2^ = 0.0098, *p* = 0.001). Although significant, the proportion of explained variance was low (0.98%), indicating that elevation alone accounts for only a small fraction of the overall compositional variation. We observed an extremely low stress value (0.002) in non‐metric multidimensional scaling (NMDS) ordination analysis indicating an excellent representation of dissimilarities in two dimensions (Figure 4). Ordination space showed a gradual compositional shift in lizard species richness along the elevational gradient, with overlapping elevation‐band ellipses suggesting partial compositional continuity rather than sharp community boundaries.

**FIGURE 5:**
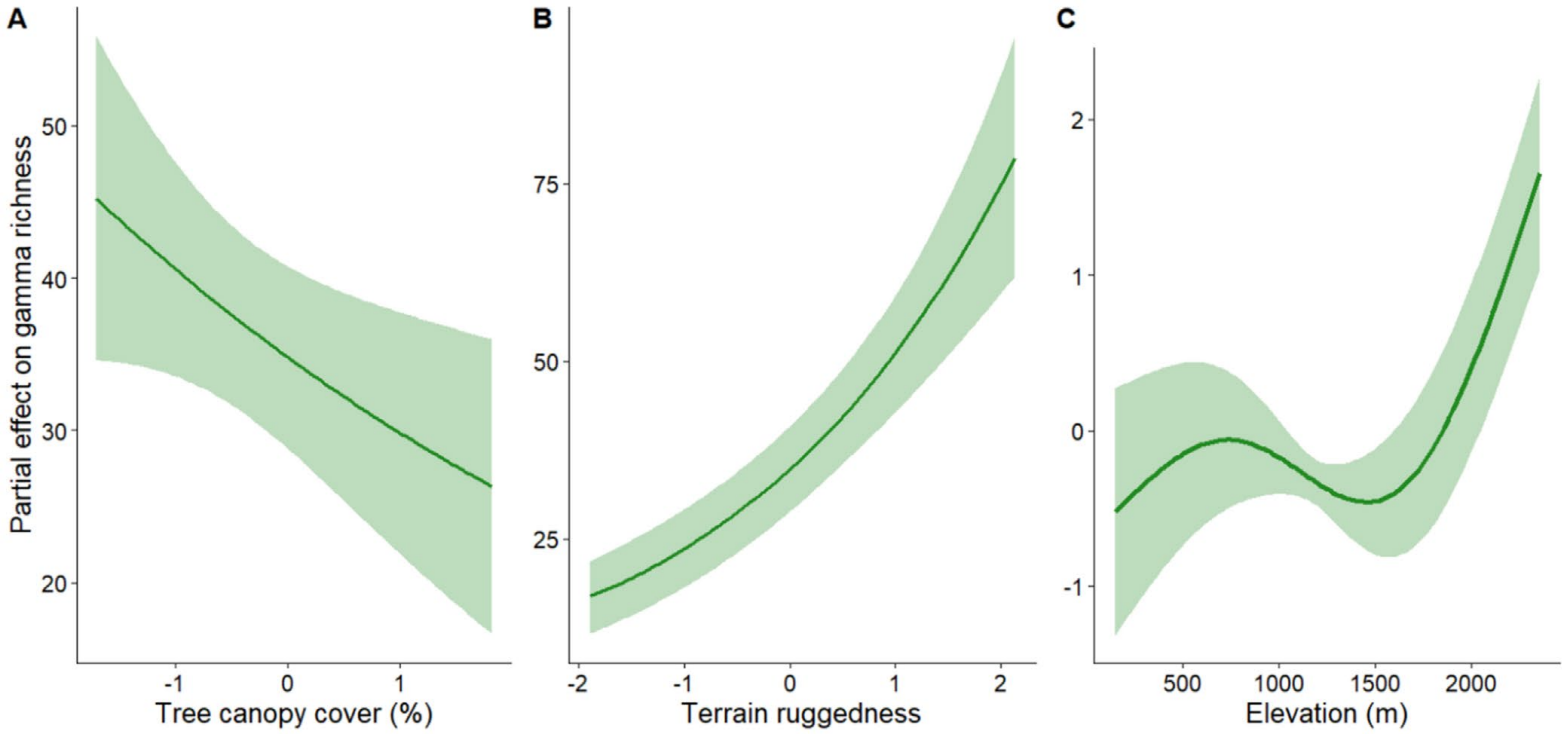
Partial response curves showing non‐linear response of lizard species richness to terrain ruggedness, tree canopy cover, and elevation.

Total (gamma) richness calculated within elevation bands exhibited variation across the gradient. Generalized additive modeling of gamma richness across elevation bands revealed a highly significant non‐linear effect of elevation (df = 2.92, *χ* ^2^ = 82.78, *p* < 0.001), explaining 85.5% of the deviance. The smooth term indicated a distinctly non‐linear elevational pattern rather than a simple monotonic increase or decline in richness. Terrain ruggedness index exerted a strong positive influence on gamma richness (*β* = 0.383, *p* < 0.001), indicating that richness increased substantially with increasing topographic complexity (Table 3, Figure 5). In contrast, we observed a significant negative impact of tree canopy cover effect (*β* = −0.154, *p* = 0.033), suggesting reduced gamma richness in areas with denser canopy structure. Soil moisture showed a marginally negative association (*β* = −0.106, *p* = 0.066). Model diagnostics indicated stable convergence and adequate smoothness selection (k‐index = 1.34), suggesting that the chosen basis dimension sufficiently captured the underlying elevational pattern without evidence of underfitting.

**TABLE 3:** Effect of factors on gamma richness of lizards across elevation bands.

Predictor	Estimate	Std. Error	*z*	*p*
Intercept	**0.223**	**0.035**	**6.378**	**< 0.001**
Tree canopy	**−0.154**	**0.072**	**−2.135**	**0.033**
Shrub cover	0.058	0.055	1.051	0.293
IAPs cover	0.042	0.067	0.625	0.532
Terrain ruggedness	**0.383**	**0.050**	**7.663**	**< 0.001**
Precipitation of driest month	0.002	0.059	0.041	0.968
Minimum temperature of the coldest month	−0.118	0.226	−0.523	0.601
Soil moisture	−0.106	0.057	−1.841	0.066

*Note:* Bold values represent predictors with significant association with gamma richness.

Partial response curves of richness showed a positive association with terrain ruggedness, a declining trend with increasing tree canopy cover, and a pronounced non‐linear response to elevation when other predictors were held constant.

## 4 Discussion

Our study provides the first ever systematic assessment of lizard species assemblage along the elevational gradient in the Nepal Himalayas. It highlights distinct environmental correlates shaping the occurrence of lizard species across the CHAL, a transition zone between lowland and highland ecosystems in central Nepal. We used descriptive statistics, GAM, and multivariate analysis which highlighted a non‐linear effect of elevation on lizard species richness. The species richness pattern as highlighted by the Spearman's rank correlation suggests that the lizard species richness does not increase or decline in a linear manner but follows a more complex non‐linear pattern. The weak monotonicity corresponds with montane environments where the distribution of species is often affected by multiple factors including but not limited to microclimate, habitat heterogeneity, and human activities rather than solely by elevation (Frey et al. 2016; Yangiboyev et al. 2024). Such non‐linear and multi‐faceted patterns emphasize the importance of ecotonal zones in maintaining and balancing species richness and underscore the ecological intricacy of reptile communities (Madani et al. 2024) in the Himalayas.

Total richness within elevation bands also showed variation across the elevational gradient. Beta diversity partitioning using Sorensen dissimilarity highlighted that the total beta diversity was almost entirely driven by species turnover and the nestedness was negligible. This shows that the compositional differences in lizard communities are mainly driven by species replacement rather than ordered species gain or loss along the gradient. Such a pattern indicates the strong effect of environmental filtering and elevational stratification where distinct assemblages occupy different elevational niches without following any kind of nested pattern (Frishkoff et al. 2019; Skeels 2020). These results align with previous studies in montane systems where turnover usually dominates the beta diversity as a result of heterogeneous microclimates, substrate conditions as well as vegetation structure across elevational gradients (Frishkoff et al. 2019; Barczyk et al. 2023). This notable turnover emphasizes that rather than focusing solely on high or low elevation zones, conservation planning in CHAL must consider preservation of habitats' heterogeneity found in different elevation bands, to sustain the full spectrum of lizard diversity.

Although PERMANOVA showed a statistically significant effect of elevation on community composition, the variation explained was relatively small. This indicates that elevation alone is a weak predictor of compositional variation, making it important to interpret the elevational effect carefully. This is probably due to lizards' composition being heavily influenced by complex ecological mechanisms such as the thermal quality of microhabitats in a restricted area, the availability of shelters, predation, and food availability (Adolph 1990; Garda et al. 2013). This might be due to local microhabitat features and site‐specific ecological requirements being dominant predictors (Adolph 1990; Garda et al. 2013). We observed an extremely low stress value in NMDS ordination suggesting an excellent representation of compositional dissimilarities. The gradual shifts in species community along elevation with overlapping elevation band ellipses indicate partial continuity rather than sharp boundaries.

We found that among the predictors employed, terrain ruggedness had a significant impact on gamma richness. This highlights the role of topographic complexity in providing lizards with access to diverse microhabitats, refuges, and foraging sites (Pal et al. 2010; de Souza‐Oliveira et al. 2025). The rugged areas are likely to provide diverse thermal microhabitats, stable surfaces, and solar radiation (Vivas et al. 2019; Žagar et al. 2023). These habitats allow lizards to warm themselves on sunny rocks and cool themselves in the shade, providing lizards with an opportunity to thermoregulate. Additionally, complex habitats provide multiple prey species (e.g., arthropods, small invertebrates) that produce high quality foraging habitat conditions in rugged areas (Adams and Gifford 2020; Chang and Todd 2023). Furthermore, rugged topography creates opportunities for a variety of species assemblages due to the presence of habitat refugia and predator escape routes (Cooper and Wilson 2007; Cooper and Whiting 2007).

Conversely, lizard species richness was negatively impacted by canopy cover, albeit weakly, suggesting that densely covered areas might not be as advantageous for lizard communities. This is consistent with the thermal ecology of ectotherms, where body temperature regulation depends strongly on external heat sources. Since thermoregulation in ectotherms involves basking, a closed canopy could limit the amount of solar radiation that reaches the forest floor (Ballinger et al. 1970; Shoemaker and Gibbs 2010). The inability to thermoregulate effectively renders the lizards more vulnerable to predators due to lower performance and possibly prolonged exposure to predation when searching for appropriate thermal conditions (Adolph 1990; Basson et al. 2017). A closed canopy can affect how much solar radiation gets to the ground and regulate the temperature of the forest floor as a result (Algar et al. 2018). Heliothermic species rely on direct sunlight to help them maintain their body temperature to assist in digestion, reproduction, and movement (Vitt et al. 1997; Qu et al. 2011). Therefore, heliothermic lizards will be affected by dense canopy that blocks sunlight.

The microclimate of an area may be altered by a closed canopy and result in increased humidity and less variation in temperature at the forest floor level. Closed canopy could result in the reduction of thermophilic species or increase the availability of species that inhabit areas of cooler temperatures (Becker et al. 2012; De Frenne et al. 2013; Jiménez‐Pérez et al. 2019). In contrast, an area that has open or partially shaded canopies provides favorable environments to animals that rely on sunlight and animals that can tolerate sunlight with an array of light and temperature conditions (Hertz et al. 1994; Kerr and Bull 2004; Pike et al. 2011). In addition, the availability of prey to forest floor inhabitants may be influenced by the structure of the forest canopy (Halaj et al. 2000; Pike et al. 2011).

Although we included broad‐scale climatic predictors in our model, they were insignificant. This suggests that long‐term macroclimatic conditions might not capture fine‐scale microhabitat‐level thermal heterogeneity experienced by lizards (Garda et al. 2013; de Souza‐Oliveira et al. 2025). The observed non‐linear increase in gamma diversity with elevation likely reflects the combined effects of habitat heterogeneity, species turnover, and microclimatic variation along elevation gradients (Bars‐Closel et al. 2017; Rosas‐Espinoza et al. 2024). This is further supported by increasing gamma diversity with increasing terrain ruggedness and decreasing canopy cover. Such complex responses are typical in montane systems where multiple ecological filters operate simultaneously (Skeels et al. 2020; Kypraios‐Skrekas et al. 2026). The absence of fine‐scale thermal measurements likely influences the strength and interpretation of observed elevational richness patterns. As a result, the inferred role of temperature in structuring lizard diversity along elevation should be interpreted cautiously.

Thermal microhabitat conditions are crucial in determining lizard distribution and activity patterns (Basson et al. 2017; Kypraios‐Skrekas et al. 2026). Hence, one important limitation of our study is the use of large‐scale bioclimatic data which may not capture the full extent of fine‐scale thermal environments experienced by lizards. Many species regulate body temperature in accordance with behavior, and species may get influenced by microhabitat conditions like ground temperature, solar exposure, and substrate types (Adolph 1990; Basson et al. 2017). Future studies incorporating microclimatic conditions will provide more precise insights on the effects of temperature or other local factors for the distribution of the lizards. Additionally, each transect was surveyed for a standardized 30‐min period; lizard detection may vary substantially across the day, with early morning and late afternoons being more favorable than midday hours. This temporal variation should be explicitly incorporated by future studies either by incorporating time of the day effects or through repeated surveys across different periods of the day.

## 5 Conclusion

This study recorded 23 species of lizards, with some species found in highlands and some in lowlands in 2018–2019. We have shown that climatic conditions and microhabitat characteristics are important factors in lizard occurrence and community structure. Species richness exhibited a non‐linear response to elevation, with localized differences among elevational bands. This reflects spatially variable richness influenced by habitat heterogeneity and species‐specific ecological requirements. Beta diversity was dominated by turnover, which indicates that community differences along elevation are primarily driven by species replacement rather than nested gain or loss of species. The study suggests that lizard diversity in CHAL is structured by habitat heterogeneity, species turnover, and elevation, and emphasizes that fragmented landscapes are equally important to lizards as much as forest and natural areas. These findings provide critical baseline data for biodiversity monitoring and offer guidance for conservation planning. Further detail and long‐term study are needed to develop more detailed conservation initiatives and fulfill knowledge gaps of lizards in the Himalayan countries like Nepal.

## Author Contributions

**Bishnu Prasad Bhattarai:** conceptualization (equal), data curation (equal), formal analysis (equal), funding acquisition (equal), investigation (equal), methodology (equal), project administration (equal), validation (equal), visualization (equal), writing – original draft (equal), writing – review and editing (equal). **Sandeep Regmi:** data curation (equal), formal analysis (equal), methodology (equal), software (equal), supervision (equal), validation (equal), visualization (equal), writing – original draft (equal), writing – review and editing (equal). **Bishnu Aryal:** data curation (equal), writing – review and editing (equal). **Pradip Kandel:** data curation (equal), writing – review and editing (equal). **Jagan Nath Adhikari:** investigation (equal), methodology (equal), writing – review and editing (equal).

## Funding

The project was partially supported by the Nepal Academy of Science and Technology, Kathmandu, and Idea Wild supported the instruments.

## Conflicts of Interest

The authors declare no conflicts of interest.

## Supporting information


Data S1:

## Data Availability

All data collected and analyzed during the study are included in the Tables, Figures, and Supporting Information of this manuscript.
